# Medication administration errors in the domain of infusion therapy in intensive care units: a survey study among nurses

**DOI:** 10.1186/s13690-023-01041-2

**Published:** 2023-02-15

**Authors:** Charlotte Beaudart, Maureen Witjes, Paul Rood, Mickael Hiligsmann

**Affiliations:** 1 Department of Health Services Research, Care and Public Health Research Institute (CAPHRI), Faculty of Health, Medicine and Life Sciences, Maastricht University, P.O. Box 616, 6200 MD Maastricht, The Netherlands; 2 Faculty of Health, Medicine and Life Sciences, Maastricht University, P.O. Box 616, 6200 MD Maastricht, The Netherlands; 3 Dutch Professional Nurses Organisation, Chapter Critical Care Nurses (V&VN IC), Utrecht, the Netherlands; 4 School of Health Studies, Research Department of Emergency and Critical Care, HAN University of Applied Sciences, Nijmegen, the Netherlands; 5 Department of Intensive Care Medicine, Radboud University Medical Center, Nijmegen, The Netherlands

**Keywords:** Infusion therapy, Medication errors, Medication administration errors, (Smart) infusion pump, Nurses, Perception, Intensive care unit

## Abstract

**Background:**

Despite extensive research carried out on medication administration errors (MAEs) in the domain of infusion therapy, there is limited knowledge on nurse’s perceptions on the occurrence of MAEs during infusion therapy. Since nurses are responsible for medication preparation and administration in Dutch hospitals, it is vital to understand their perspectives on the risk factors for MAEs.

**Aim:**

The purpose of this study is to investigate the perception of nurses, working in adult ICUs, on the occurrence of MAEs during continuous infusion therapies.

**Methods:**

A digital web-based survey was distributed among 373 ICU nurses working in Dutch hospitals. The survey investigated nurses’ perceptions on the frequency, severity of consequences and preventability of MAEs, factors for the occurrence of MAEs, and infusion pump and smart infusion safety technology.

**Results:**

A total of 300 nurses started to fill out the survey but only 91 of them (30.3%) fully completed it and were included in analyses. Medication-related factors and Care professional-related factors were perceived as the two most important risk categories for the occurrence of MAEs. Important risk factors contributing to the occurrence of MAEs included high patient-nurse ratio, problems in communication between caregivers, frequent staff changes and transfers of care, and no/incorrect dosage/concentration on labels. Drug library was reported as the most important infusion pump feature and both Bar Code Medication Administration (BCMA) and medical device connectivity as the two most important smart infusion safety technologies. Nurses perceived the majority of MAEs as preventable.

**Conclusions:**

Based on ICU nurses' perceptions, the present study suggests that strategies to reduce MAEs in these units should focus on, among other factors, the high patient-to-nurse ratio, problems in communication between nurses, frequent staff changes and transfers of care, and no/incorrect dosage/concentration on drug labels.

**Supplementary Information:**

The online version contains supplementary material available at 10.1186/s13690-023-01041-2.

## Introduction & background

Worldwide, medication errors are a major cause of injury and avoidable harm in healthcare with estimated costs of 42 billion US Dollars annually [1]. Medication errors are rated as one of the highest causes of death and are considered among the most common causes of morbidity and mortality in hospital settings [2, 3]. The 1999 Institute of Medicine report estimated that more than one million injuries and nearly 100,000 deaths occur annually in the US as a result of preventable mistakes in health care [4]. Medication errors occur frequently: 106 per 1,000 patient days in intensive care units (ICUs) of Dutch hospitals [5]. About 2% of medication errors result in significant patient harm (e.g. injury, prolonged hospital stay, death) [6]. The occurrence of medication administration errors (MAE) is higher in the ICU compared to other wards of the hospital, and occur most frequently during the medication administration phase [7, 8]. Patients in the ICUs are critically ill and receive more medication compared to patients on other hospital wards. Furthermore, high risk medication with narrow therapeutic indexes, and the high frequency in which these are administered makes critically ill patients particularly vulnerable to injury from medication errors [7, 9]. Among medication errors, intravenous errors are very prevalent. In a systematic review of UK studies, errors were found to be five times more likely in intravenous than non-intravenous doses [10]. An international systematic review estimated the probability of making at least one error in the preparation and administration of a dose of IV medication to be 73% [11]. Thereby, intravenous medication administrations pose higher risks and severity of error compared to other medication administration routes [12, 13]. The administration of IV medication has been identified as a significant topic of concern by regulators, manufacturers, and health-care providers [14].

Medication administration with infusion therapy is a complex process. Indeed, it involves a multiple adaptive system with multiple interacting agents, such as professionals, patients, software systems, which can highly interact [15, 16]. In 2019, Wolf et al. published a report concerning best practices to decrease infusion-associated medication errors. In this report authors presented a large series of recommendations/strategies that may be applied [17]. Technologies, such as smart pumps which are medication delivery devices that use a combination of computer technology and drug libraries, have been developed to reduce the incidence of intravenous errors. The features of infusion pumps are focused on improving the accuracy of intravenous infusions and enabling healthcare professionals to program their flow, volume, and timing [18]. New infusion pumps, so-called smart pumps, can have predetermined clinical guidelines, *Dose Error Reduction Systems* (DERS), and drug libraries [18]. Also, smart pump technology can have features such as wireless connectivity and error reporting. Even if some research has suggested that intravenous smart pumps may have the potential to reduce MAEs [16, 19], evidence regarding the direct impact of development of those types of technologies on the reduction of MAEs is mixed [16, 20–22]. Indeed, many factors other than devices themselves have been reported as affecting infusion administration [15, 22].

Despite extensive research on the prevalence and risk of MAEs in the field of infusion therapy, there is limited knowledge about nurses' perceptions of the occurrence and risk factors of MAEs during infusion therapy. As nurses are responsible for the preparation and administration of medication in Dutch hospitals, it is important to understand their perspective on the risk factors for MAEs. Measuring the perceptions of first-line healthcare professionals may lead to the identification of new risk factors. Strategies to reduce MAEs in the ICU could be developed accordingly.

Therefore the objective of the present study is to investigate the perception of nurses working in the Dutch adult ICUs on the occurrence of MAEs during infusion therapy and the use of infusion pumps and smart infusion safety technology. More specifically, the following three research questions are addressed: (1) What is the perception of nurses on the frequency, severity, and preventability of different types of MAEs that occur during infusion therapy? (2) What is the perception of nurses on risk factors associated with committing MAEs during infusion therapy? (3) What is the perception of nurses on the impact of infusion pump features and smart infusion safety technology on committing MAEs?

## Methods

A cross-sectional descriptive study design was used. A digital, web-based survey was developed and executed among nurses working in adult ICUs in Dutch hospitals. The Consensus-based Checklist for Reporting of Survey Studies (CROSS) [23] was used as a tool for reporting the survey study.

### Definition

This study research focused on medication errors, defined by the National Coordinating Committee’s Medication Error Reduction Programme (NCC-MERP) as “*any preventable event that may cause or lead to inappropriate medication use or patient harm while the medication is being controlled by the health care professional, patient, or consumer. Such events may be related to professional practice, health care products, procedures, and systems, including prescribing, order communication, product labeling, packaging, and nomenclature, compounding, dispensing, distribution, administration, education, monitoring, and use* [24]”. This safety outcome therefore differs from effectiveness outcomes such as adverse drug reactions or adverse drug events.

### Survey development

The survey was developed using topic-related studies [25, 26] and optimized interviews with three experts (a researcher in the field of the research topic, a hospital pharmacist, and an ICU nurse) which assessed the relevance, difficulty, and appropriateness of the questions. Based on their expertise, the experts added several items tailored to the current daily practice in the Netherlands. The final version of the survey was pilot tested by two ICU nurses.

The final survey consisted of six parts. The first part consisted of demographic and medical characteristics such as age, gender, type of hospital and ICU size. The second part assessed MAEs at ICU: perceived frequency of occurrence, perceived severity of consequences, and perceived preventability of the errors. A total of 17 types of MAEs were assessed. The third part measured the importance of (risk)factors in six categories for the occurrence of MAEs (a total list of 38 factors related to medication, patient, intensive care, care professional, shifts, and the infusion pump). A seven-point Likert scale was used to measure the perceived importance of the (risk) factors (1 = very unimportant, 4 = neutral, 7 = very important) [27]. The fourth part measured the importance of eleven infusion pump features for reducing MAEs (also using seven-point Likert scales). The fifth part focused on the use of smart infusion safety technology and their impact on medication safety. The last part included questions on reporting errors and the importance for minimizing medication administration errors (Appendix A).

### Study population

The study population of the research study contained nurses working in the adult ICU in hospitals located in the Netherlands. There was no restriction on the setting in which nurses could work, as long as they were working in an intensive care setting. Inclusion criteria were (1) ICU nurses, (2) 18 years and older, (3), working in any type of Dutch hospital in the intensive care setting and (4) Dutch or English speaking. There were no requirements about the years of work experience. Exclusion criteria for the respondents were (1) interns or students, (2) non-Dutch or non-English speaking employees.

### Survey administration

Data collection took place between May 2021 and September 2021. The survey was executed using the online survey platform Qualtrics (www.qualtrics.com). Convenience sampling and snow-ball sampling were applied to the study population [28, 29]. Prior to the survey distribution, connections were built up and optimized to create a strong network. Online survey invitations were spread via e-mail and social network platform LinkedIn among the network of the researchers consisting of ICU nurses working in various types of hospitals in the Netherlands. Contacts were requested to forward the survey invitation to their connections and colleagues. The mailings were followed up by a reminder one week after the first mailing. In addition, hospitals and nurses’ associations were approached to advertise the survey invitation. Furthermore, a survey invitation was included in the monthly national newsletter of the critical care nurses’ chapter of the Dutch Professional Nurses Organisation, and in the local newsletters of the ICU of two Dutch hospitals.

### Analysis

Only respondents who finished the whole survey and filled out all mandatory questions were included for analysis. Descriptive statistical analysis on the demographic data was carried out, containing distribution of frequencies, mean, standard deviation (SD), median and interquartile range (P25-P75). The theoretical framework of Reasons (1990) to describe human error was considered to classify risk factors [30]. Five categories of factors were further identified: medication-related factors, patient-related factors, environmental factors (i.e. intensive care-related factors), professional-related factors and infusion-pump-related factors. Correlations were measured between those five categories using Spearson correlations and parametric statistics for measuring mean differences were applied to measure significant differences between categories. Moreover, differences across sub-groups about the perceived importance of risk factors were calculated using Student T test for variables binarized into two categories (age < 45 years or ≥ 45 years, categorized by the median age of the sample; sex women or men; years of experience < 15 years or ≥ 15 years categorized by the median years of the sample; and number of beds 0–20 or ≥ 20 beds) and using ANOVA test for type of hospital (academic vs general vs top clinic).

All analyses were carried out using IBM SPSS software Version 27.

### Ethics

Ethical approval was obtained at Maastricht University (number 2021.045). At the start of the survey, respondents had to give their informed consent for participation in the study to protect the safety and privacy of the respondents participating in the survey study. All collected data were anonymous.

## Results

### Respondents

An estimate of 373 intensive care nurses were approached using the online survey invitation. More IC nurses may have been exposed to the survey invitation via the newsletter and snowball techniques, but that number cannot be estimated correctly. A total of 300 nurses started to fill out the survey, of which 209 nurses did not fully complete the survey. A total of 91 nurses fully completed the survey and were included in the study sample and data analysis. Duration of the questionnaire was 24 ± 18.3 min.

The final sample consisted of 53 women (58.2%) and 38 men (41.8%), with an average age of 43.8 ± 11.7 years (ranging between 25 and 65 years). Most nurses worked in general hospitals (50.5%), followed by academic hospitals (24.1%) and top-clinical hospitals (24.2%). Work experience ranged from one to 41 years, with a mean of 16.7 ± 11.2 years. Size of ICUs where nurses worked were mainly 11–20 beds (35.2%) and 21–30 beds (26.4%), followed by 31–40 beds (20.9%), 0–10 beds (11.0%) and the least number of nurses worked in ICUs sized 41–50 beds (6.6%) (Table with characteristics available in Appendix B).

### Frequency, severity, and preventability of different types of MAEs

**Fig. 1:**
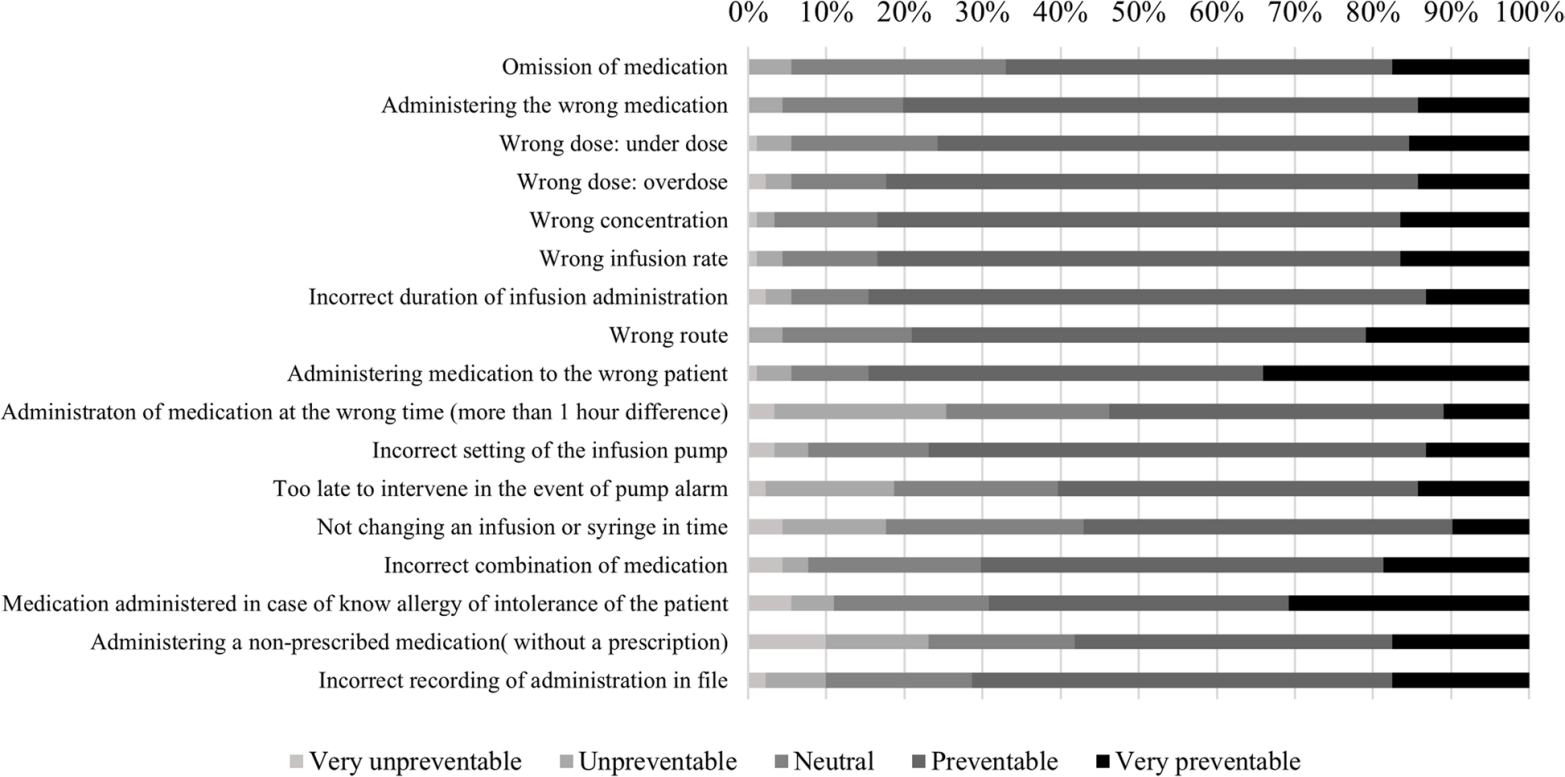
Perceived frequency of types of MAEs

Most nurses recognized the occurrence of most MAE types. The three more common MAEs were *administration of medication at the wrong time (*perceived as type of MAE that happens daily by 18.7%), *administrating a non-prescribed medication* (perceived as type of MAE that happens daily by 16%) and *wrong infusion rate* (perceived as type of MAE that daily happens by 14.3%). The three less common MAEs were *administering medication to the wrong patient* (perceived as type of MAE that never happens by 29.7%) and *too late to intervene in the event of pump alarm* as well as *medication administered in case of known allergy* (perceived as types of MAEs that never happens by 23.1%) (Fig. 1).

**Fig. 2:**
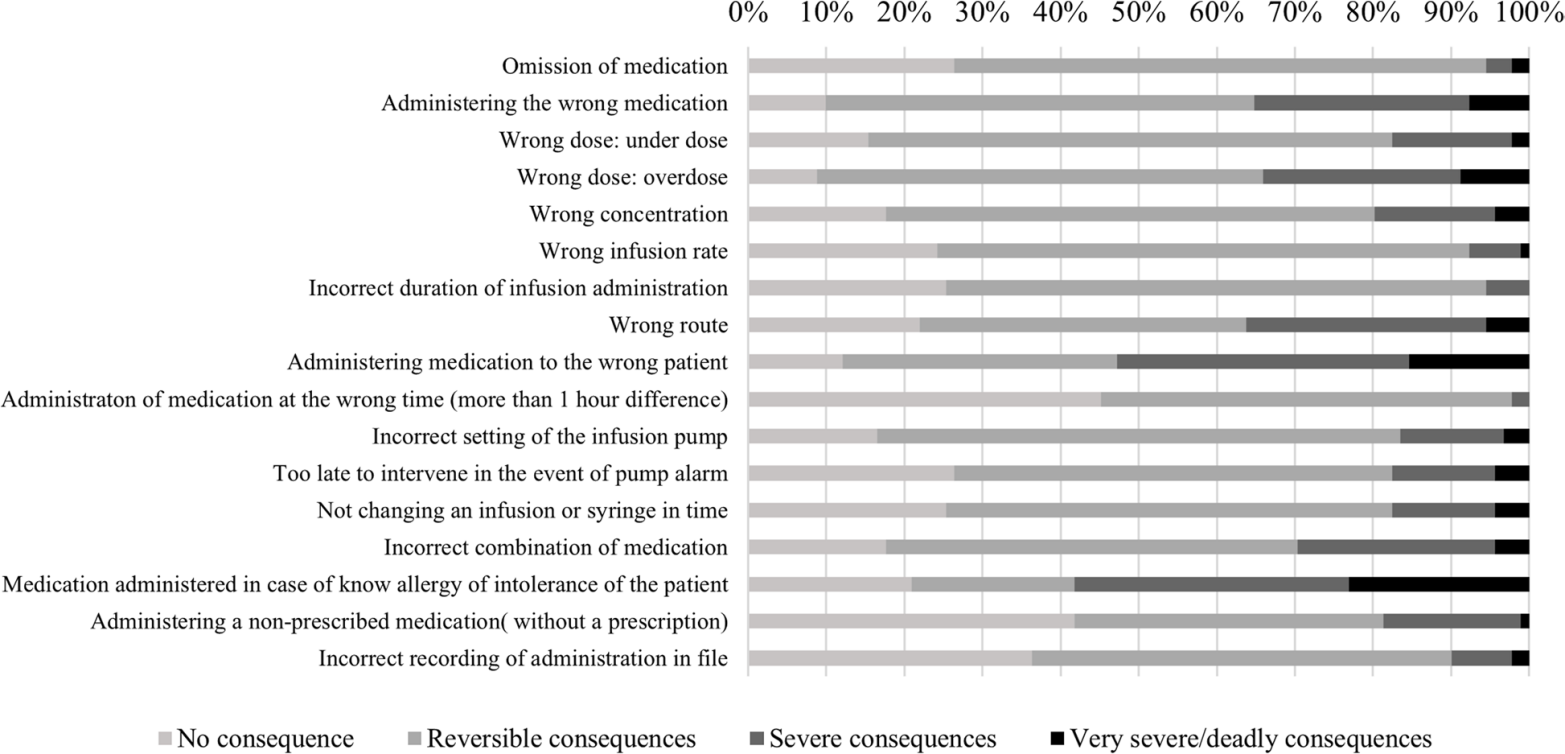
Perceived severity of consequences of types of MAEs

Regarding consequences, nurses perceived *medication administered in case of known allergy or intolerance of the patient* as the most severe type of MAE, followed by *administration medication to the wrong patient*, with respectively 58.3% and 52.8% of nurses scoring “severe” or “very severe/deadly consequences”. *Administration of medication at the wrong time* is perceived by most nurses as type of MAE resulting in no consequences (45.1%) (Fig. 2).

Looking at preventability, all types of MAEs were considered more as “preventable” than “non preventable”.

**Fig. 3:**
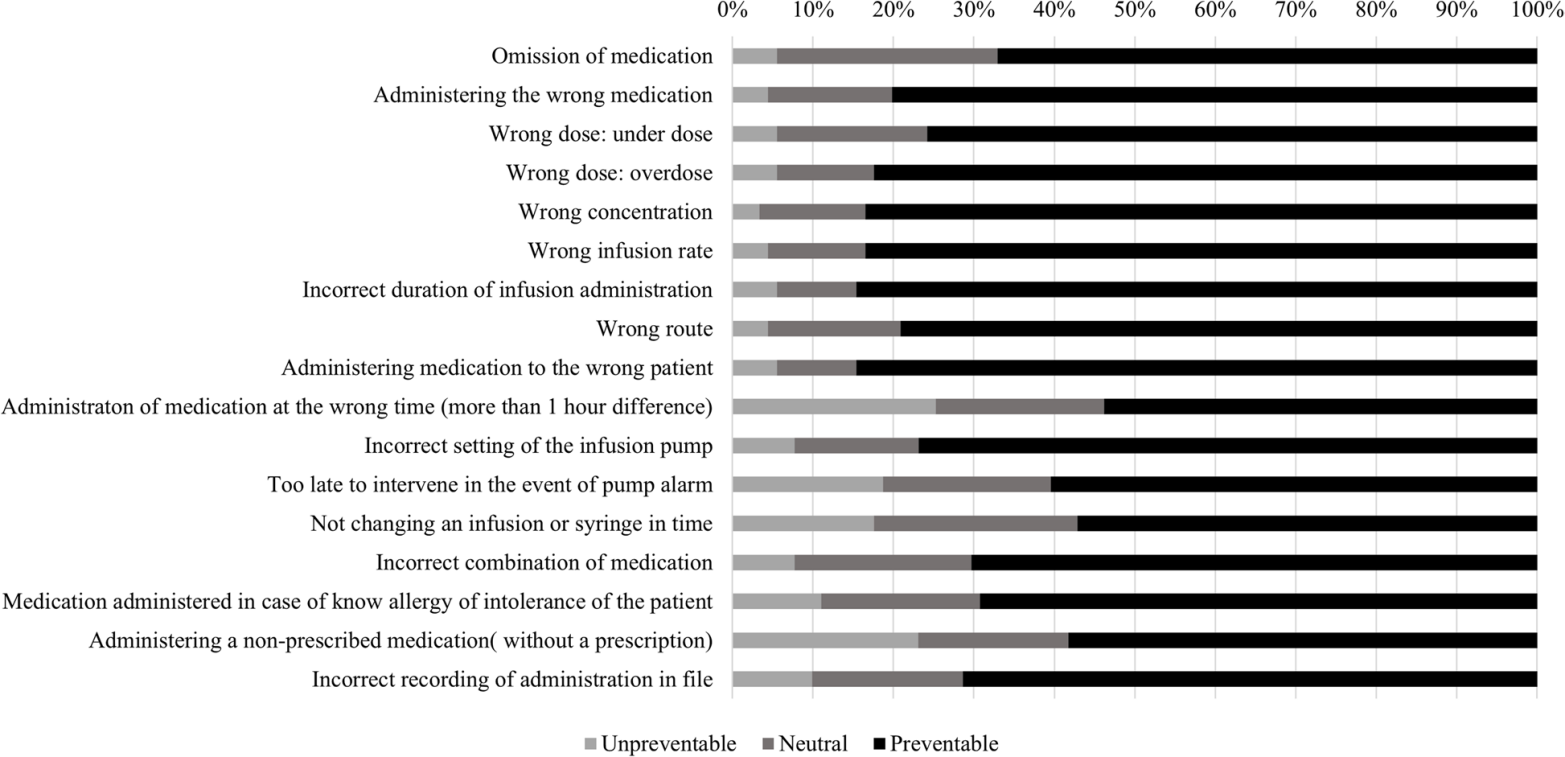
Perceived preventability of types of MAEs

Out of all types of MAEs*, administering medication to the wrong patient* and *incorrect duration of infusion administration* were perceived as the two most preventable MAEs, with most nurses scoring the MAE as “preventable” (84.6%). On the contrary, *administration of medication at the wrong time* was considered as the most unpreventable type of MAE with 25.3% of nurses perceiving this MAE as “unpreventable” (Fig. 3).

### (Risk) factors associated with committing medication administration errors during infusion therapy

(Risk) factors associated with committing MAEs during infusion therapy were reported by category. All features appeared to be important for participants (mean range from 3.45 to 5.26 points on a scale from one to seven points). The three most important factors’ categories were, in order of importance, care professional-related factors, medication-related factors and intensive care-related factors. The median of those three categories does not significantly differ between each other’s but significantly differ from the two other categories, considered less important by the sample (i.e. patient-related factors and infusion pump-related factors, *p* < 0.001). All categories correlated significantly with the others, indicating that perception of the importance of risk factors seems homogeneous regarding the categories of risks factors (Matrix of correlation reported in Appendix C).

Within the care professional-related factors category (mean of 5.26 ± 0.99 on one to seven Likert scale of importance), *problems in communication between caregivers* was on average considered as the most important (risk) factor (5.74 ± 1.36) and *Insufficient information and/or knowledge about using the pump* was perceived as the least important care professional-related factor (4.26 ± 1.61). Within the medication-related factors (mean of 5.17 ± 1.16 points of importance), *no or incorrect dosage/concentration on labels* was the most important factor within the medication-related factors (5.62 ± 1.59), followed by *large amount of medication* (5.02 ± 1.45). *Type of medication (effect*) was scored as the least important within the category (5.02 ± 1.59). Within the intensive-care related factors (mean of 5.00 ± 0.82), nurses ranked *patient-nurse ratio on the ward* as the most important factor contributing to the occurrence of MAEs (5.89 ± 1.43). This factor was followed by *frequent staff changes and frequent transfers of care* (5.80 ± 1.29) and *urgent admissions* (5.76 ± 1.37). *Early or late shift* of caregivers was generally perceived as the least important intensive care-related factor of this category (3.75 ± 1.30 and 3.69 ± 1.32 respectively). Within the patient-related factors (mean of 4.53 ± 1.23), *large number of treatments/interventions* was considered most important (5.56 ± 1.65) and *sedation of patients* (patients unable to participate in care) was considered least important (3.80 ± 1.65). Infusion pump-related factors (mean of 4.21 ± 1.32 points of importance) was considered the least important of all categories (Table 1).

**Table 1:** Perceived importance of (risk) factors contributing to MAEs: descriptive statistics on 7-point Likert scale scores

	**Mean ± SD**	**Median (P25-P75)**
**Care professional-related factors**	**5.26 ± 0.99‡**	**5.35 (4.57–5.89)**
Problems in communication between caregivers	5.74 ± 1.36	6.00 (5.00–7.00)
Mental state/sleep deprivation/fatigue	5.67 ± 1.18	6.00 (5.00–7.00)
Notation errors in file	5.46 ± 1.38	5.00 (4.00–6.00)
Shortage of medication knowledge	5.40 ± 1.36	6.00 (5.00–6.00)
Wrong reading of the file	5.18 ± 1.39	5.00 (4.00–6.00)
Shortage of work experience	4.95 ± 1.41	5.00 (4.00–6.00)
Insufficient information and/or knowledge about using the pump	4.41 ± 1.63	4.00 (3.00–6.00)
**Medication-related factors**	**5.17 ± 1.16¶**	**5.33 (4.33–6.00)**
No or incorrect dosage/concentration on labels	5.62 ± 1.45	6.00 (5.00–7.00)
Large amount of medication	5.02 ± 1.59	5.00 (4.00–6.00)
Type of medication (effect)	4.87 ± 1.74	5.00 (4.00–6.00)
**Environmental factors—Intensive care-related factors**	**5.00 ± 0.82†**	**5.00 (4.44–5.53)**
Patient-nurse ratio on the ward	5.89 ± 1.43	6.00 (5.00–7.00)
Frequent staff changes and frequent transfers of care	5.80 ± 1.29	6.00 (5.00–7.00)
Urgent admissions	5.76 ± 1.37	6.00 (5.00–7.00)
Deviation from protocols / hospital policy	5.68 ± 1.20	6.00 (5.00–7.00)
Multiple different caregivers working	5.40 ± 1.34	5.00 (5.00–7.00)
Complex environment	5.40 ± 1.65	6.00 (4.00–7.00)
Use of new technologies and treatments	5.16 ± 1.42	5.00 (4.00–6.00)
Shifts: night shift	5.09 ± 1.51	5.00 (4.00–6.00)
Premature and nocturnal discharge	4.32 ± 1.41	4.00 (4.00–5.00)
Insufficient supervision of the caregivers	4.26 ± 1.61	4.00 (3.00–5.00)
Shifts: early shift	3.75 ± 1.30	4.00 (3.00–4.00)
Shifts: late shift	3.69 ± 1.32	4.00 (3.00–4.00)
**Patient-related factors**	**4.53 ± 1.23§**	**4.67 (3.67–5.33)**
Large number of treatments/interventions	5.56 ± 1.65	6.00 (5.00–7.00)
Severity of the disease	4.88 ± 1.91	5.00 (4.00–7.00)
Patient agitation	4.43 ± 1.58	5.00 (3.00–5.00)
Lack of usual medication list (home medication)	4.30 ± 1.69	4.00 (3.00–5.00)
Long-term hospitalizations	4.19 ± 1.70	4.00 (3.00–5.00)
Sedation: patients unable to participate in care and defend themselves against mistakes	3.80 ± 1.65	4.00 (3.00–5.00)
**Infusion pump-related factors**	**4.21 ± 1.32**	**4.12 (3.50–5.12)**
Problem in the pre-administration process	4.79 ± 1.58	5.00 (4.00–6.00)
Hoses/connections	4.63 ± 1.62	5.00 (4.00–6.00)
Low user-friendliness	4.51 ± 1.85	4.00 (3.00–6.00)
Snapping the correct syringe into the pump	4.37 ± 1.90	4.00 (3.00–6.00)
Programming/choosing the right pump (software)	4.32 ± 1.67	4.00 (3.00–6.00)
Infusion pump failure	3.99 ± 1.68	4.00 (3.00–5.00)
Device maintenance	3.64 ± 1.78	4.00 (2.00–5.00)
Insufficient presence of required characteristics of the pump	3.45 ± 1.70	4.00 (2.00–4.00)

^‡^significantly different from patient-related factors and infusion pump-related factors (both *p*-values < 0.001)

^¶^significantly different from patient-related factors and infusion pump-related factors (both *p*-values < 0.001)

^†^significantly different from patient-related factors and infusion pump-related factors (both *p*-values < 0.001)

^§^significantly different from care-professional-related factors, medication related-factors, patient-related factors (all *p*-values < 0.001) and infusion pump-related factors (*p* = 0.01)

Subgroup analyses revealed no factors influencing the perceived importance of risk factors, with one exception. In fact, there was a significant difference in the importance placed on infusion pump-related factors by hospital type, with academic hospitals having lower importance compared to general hospitals and top clinics (*p* < 0.01) (Appendix D).

### Infusion pump features and smart infusion safety technology

Availability of infusion pump features on the ward was measured to indicate the familiarity of nurses with the features. Again, all features appeared to be important for participants (mean range from 4.36 to 5.58 points on a scale from 1 to 7 points). Four (4.4%) nurses indicated that there was a *smart pump with DERS* on the ward and 19.8% of nurses did not know about its availability. Results on availability showed that *drug library* was available on the majority of wards (*n* = 82, 90.1%). Furthermore, most available infusion pump features were *the ability to set highly accurate occlusion parameters* (*n* = 57, 62.6%) and *pre-programmed protocols for the concentration/dilution ratios* (*n* = 48, 52.7%). Average Likert scale scores (mean) and SD of the perceived importance of infusion pump features for the reduction of medication errors in the drug administration phase are displayed in Table 2. On average, *drug library* was perceived as the most important infusion pump feature (5.58 ± 1.57). Among all the pump features, *the ability to remotely monitor and modify the infusion* was rated as the least important one but was nevertheless rated with a mean importance of 4.36 ± 1.68 out of 7 points.

**Table 2:** Perceived importance of infusion pump features and smart infusion safety technology

	**Mean ± SD**	**Median (P25-P75)**
**Infusion pump features**	4.72 ± 1.69	
Drug library	5.58 ± 1.57	6.00 (5.00–7.00)
Ability to load the syringe (or bag) and set the infusion parameters quickly in case of emergencies	5.21 ± 1.63	6.00 (4.00–6.50)
Pre-programmed protocols for the concentration/dilution ratios	4.84 ± 1.95	5.00 (4.00–7.00)
Smart pumps with Dose Error Reduction Software (DERS)	4.81 ± 1.57	5.00 (4.00–6.00)
Infusion rate control systems	4.69 ± 1.48	5.00 (4.00–6.00)
Locking systems and alarm systems in case of wrong dose setting	4.64 ± 1.72	5.00 (4.00–6.00)
Ability to set highly accurate occlusion pressure parameters	4.53 ± 1.58	4.00 (4.00–6.00)
In the event of an emergency start, the possibility to enter all safety procedures later	4.52 ± 1.66	4.00 (4.00–6.00)
Pre-set start speed and minimum / maximum speed that considers the weight of the patient	4.44 ± 1.71	4.00 (4.00–6.00)
Pre-programmed protocols for minimum and maximum dose	4.38 ± 1.83	4.50 (3.00–6.00)
Ability to remotely monitor and modify the infusion	4.36 ± 1.91	4.00 (3.00–6.00)
**Smart infusion safety technology**	5.25 ± 1.38	
Bar Code Medication Administration (BCMA) or optical medication scanners	5.57 ± 1.54	6.00 (5.00–7.00)
Medical device connectivity (interoperability between medical devices with EHR)	5.36 ± 1.38	6.00 (5.00–6.00)
Anonymous statistical reporting for setting errors	5.16 ± 1.33	5.00 (4.00–6.00)
Electronic administration	4.90 ± 1.25	5.00 (5.00–6.00)

All the four types of smart infusion safety technology aspects were rated higher than 5.00. *Bar code Medication Administration (BCMA)* and *medical device connectivity* were perceived as the two most important smart infusion safety technologies in reducing the occurrence of MAEs (5.57 ± 1.54 and 5.36 ± 1.38, respectively). *Electronic administration* was ranked as the least important one compared to the other safety technologies (5.03 ± 1.33).

## Discussion

The present study delivers valuable insights in the perceptions of ICU nurses on committing MAEs, and particularly during infusion therapy with the infusion pump. Looking at the types of MAEs, most nurses recognized the occurrence of types of MAEs. The majority of nurses do not perceive MAEs as resulting in severe consequences and perceive MAEs mostly as preventable errors. This might implicate that they do not perceive MAEs as an event that could cause a high level of harm. This finding is similar to previous studies, which found that a high number of intravenous medication errors occur, while relatively few errors result in harm or injury [21]. However, other studies stated that medication errors in the ICU could have serious consequences because of the complex ICU setting and critical condition of the patients [31]. Regarding the results on perceived frequency of MAEs, faults in dose, concentration, route, and administration were perceived mainly as rarely (once-yearly) occurring types of MAEs. A previous study on the perception of nurses towards medication errors in the ICU in the US found similar results on the frequency of these types of MAEs [32], and suggested that faulty infusion rates, dosage, concentration of medication, routes and incorrect medication delivered due to misidentification of a patient, were perceived as rare [32]. However, wrong infusion rate was considered as a weekly occurring error by the nurses in the present research study, which makes it a more frequently perceived error than was found in the study of Mahmood, Chaudhury and Valente [32]. In its recent systematic literature review, Sutherland et al. [33] reported the frequency and nature of MAEs affecting hospitalised children. No less than 3,270 observations from three individual studies [34–36] were taken into account to highlight a general prevalence of errors of 16.3% mainly represented by technical/preparation errors (i.e. 60%), followed by wrong time administration (i.e. 32.4%) and wrong rate of administration (i.e. 25%).

In the present study, a high patient-nurse ratio was perceived as the most important risk factor for the occurrence of MAEs. This finding could be supported by a previous study conducted among Iranian nurses, where high patient-nurse ratio was perceived as the most important contributor to medication errors [37]. Besides studies performed on perception, previous research supports the high patient-nurse ratio as one of the most important factor contributing to MAEs [32, 38]. Since high patient-nurse ratios result in a higher workload for the nurses, reducing patient-nurse ratios could be considered as an important strategy by ICU for the prevention of MAEs. However, even if reducing patient-nurse ratio is an ideal, this could nevertheless be very challenging for hospitals. In the systematic literature search published by Keers et al., which includes 91 observational studies reporting MAEs data, authors highlighted that wrong-time administration errors are the most prevalent error subtypes and can be viewed as a product of systems failures such as workload [13]. Wrong administration rate and preparation errors were also reported as two of the most frequent MAEs.

Regarding the care professional-related factors, the present study found that problems in communication between caregivers showed the highest score of importance as contributor. This is also highlighted in the large observational study published by Lyons et al. Authors reported that a large proportion of MAEs involved oral orders or order changes that have not been correctly documented [34]. In addition, within medication-related factors, no or incorrect dosage/concentration on labels was perceived as an important medication-related factor. These findings are supported by a previous study conducted among nurses working in a variety of wards, which detected medication packaging and physician communication as the highest-scoring factors contributing to medication errors [39]. Similar finding was made by Mrayyan, Shishani and Al-Faouri, where they discovered that the highest perceived contributory factor of medication errors was damaged or poor quality medication labels/packaging [40]. Based on those findings, labels and packaging might be considered as an important risk factor for committing MAEs.

Our results reported that most nurses perceived the smart infusion safety technology as an important tool to reduce MAEs. In their systematic literature search published in 2014 [22] Ohashi et al. reported mixed results in regard to the efficacy of smart pumps and drug libraries. They reported that smart pumps may reduce the MAEs but will not eliminate them. There are indeed some types of error that still persist after their implementation. Acceptance, compliance, and trust are the challenges when implementing smart infusion pumps and may be contributing factors to the reduction of MAEs. Montague et al. reported that nurses’ trust in smart pumps is influenced by technology characteristics of the pump (e.g. speed of programming, reliability and durability of the pump), individual user characteristics (e.g. previous experience with technology), and organization characteristics (e.g. work environment that prevents worker fatigue) [41]. Carayon et al. [42] further studied the acceptance of a large sample of US nurses towards the smart infusion pump technology they found, as in our survey, that nurses had positive perceptions on the smart infusion pump regarding the enhancement of quality and safety. Those results may indicate that nurses are generally open towards and accepting of the use of new technologies during medication administration, which could be beneficial for the implementation of such technologies in the ICU. Importantly, Carayon et al. [42] also reported that acceptance of smart pumps increased significantly one year after implementation. Indeed, after one year of use, nurses' perception of pump efficiency positively influenced its acceptance and its use. Ongoing education after smart pump implementation may therefore be important. Among technologies, the current study shows the importance of the drug library as infusion pump feature, according to most nurses in the study sample. A previous study also revealed that the drug library was a predictor for user acceptance as it helped to prevent medication errors [42]. Taylor and Jones highlighted the importance of the drug library as a safety strategy to prevent wrong dose, wrong infusion rate, and other setting errors [43]. The use of bar code technology is also considered a very important feature in our study and is defined as the most effective barrier to prevent errors and ensure traceability in the study of Otero et al. in Spain [44]. All these results highlight the need to adopt and promote different technologies to prevent MAEs.

Several suggestions for practice can be made. First of all, we suggest hospitals to pay special attention to the high patient-nurse ratio and workload of nurses, problems in communication between caregivers, and no or incorrect dosage/concentration on labels, which were perceived as important risk factors for the occurrence for MAEs by nurses. These risk factors could be useful to address in prevention strategies. However, as previously mentioned, some of these intensive-care related actions may be very challenging for hospitals and other complementary actions are needed. Attention should therefore also be drawn to infusion pump features that were considered as important to reduce MAEs, such as smart pumps, drug libraries, automation, BCMA or medical device connectivity. Secondly, because frequent staff change has also been reported as an important potential factor of MAEs, special emphasis could also be needed on staff training and post-implementation support. Even if the present findings may not recommend prioritizing education and training on the usage of the infusion pump to nurses in the prevention of MAEs, many other studies highlighted the benefits of such technologies. Strategies to increase nurse acceptability of smart pumps, to increase adoption of smart pumps and to ensure continuous education on this technology tool should be encouraged as compliance in using smart pumps remains a key toward preventing errors [16, 22]. Moreover, the infusion pump could be considered as focus point during prevention strategies for MAEs. Thirdly, we also suggest involving nurses in prevention strategies as nurses play a vital role in reducing risks of committing MAEs. Furthermore, our findings suggest that nurses perceived the majority of MAEs as preventable and resulting in mild consequences. Therefore, we suggest increasing awareness among nurses on the risks of MAEs considering the potential harm of some MAEs. Lastly, the present study found that nurses perceived a variety of risk factors as important contributors to the occurrence of MAE, which could lead to recommending hospitals to focus on a combination of risk factors when tackling the issue of MAEs.

### Limitations

There are several limitations to the present study that need to be addressed. Firstly, due to the cross-sectional nature of this study, no causal relationship can be inferred. Secondly, even if the sample size appears adequate for the study objective, no sample size calculation was performed. In addition, we cannot guarantee that the population included is truly representative of the target population. Only volunteer participants, possibly the nurses most likely to be affected by the issue, completed the survey and therefore a volunteer bias could not be excluded. Thirdly, this study does not contain any actual data on MAEs, since only nurses’ perceptions were investigated. However, as nurses in the ICU play a key role in preparation and administration of the intravenous medications, their experiences and perceptions could provide vital information for further optimalisation of these processes and assessment of the feasibility of technical solutions for the prevention of MAEs. As a fourth limitation, we can state that our study was conducted during the COVID-19 pandemic and pressure on ICUs, may have influenced nurses’ perception and overall results. Hence, an adequate sample of responses was gathered, and as our results align with previous findings, we estimate them valid. As a fifth limitation, as a convenience sample was used, our sample may not broadly reflect the average nurse’s opinion. However, we used our national network and several sources for recruiting participants, which we expect to be sufficient to gather a valid representation of professionals in our country. Limitation number six: although we attempted to ensure the content validity of the survey by involving an ICU nurse in the development of the survey items, we did not statistically measure the content validity of the survey. This could have been done to ensure that all areas of research were covered by the survey. Limitation number seven: the question about the preventability of medication errors may have been susceptible to information bias. In fact, all errors are, by definition, preventable. By asking participants to what extent they considered some errors to be preventable or not, we may have introduced a systematic bias into the data collection. Finally, we did not collect the names of the hospitals that participated in our survey because we wanted to preserve the anonymity of the questionnaires. This choice prevents us from ensuring the representativeness of the Dutch hospital in our sample and therefore limits the external validity of our results. In the same spirit, we did not collect nurses' unit specialties. We only asked participants about the type of hospital they worked in, the size of the hospital in terms of ICU beds, and the job function (i.e., nurse or managerial position). Additional information would have been interesting to better characterize our population.

## Conclusions

In conclusion, the present study enabled further insight into the occurrence of MAEs during the intravenous medication administration process. Based on ICU nurses’ opinions, results of this survey suggest that strategies for MAEs reduction should focus on some environmental factors such as the high patient-nurse ratio in intensive care units or the frequent staff changes and frequent transfers of care. Care professional-related factors such as problems in communication between caregivers or sleep deprivation/fatigues appear to be important factors as well. Attention for medication-related factors such as the correct labelling of drugs is also suggested. Patient- related factors and infusion pump-related factors were rated as less important than the other factors. Nurses also considered that insufficient knowledge about how to use the pump is a less important risk factor for MAEs. Besides this, nurses appeared to have a relatively positive perception of the importance of drug libraries and medical device connectivity. The present study reveals how nurses perceived a broad range of risk factors as contributors to the occurrence of MAE which emphasizes that MAEs are a highly multi-caused issue in healthcare. Therefore, all types of sources of MAEs should deserve attention when developing prevention strategies for MAEs.

## Supplementary Information

**Additional file 1: Appendix A.** Survey English version (translated). **Appendix B.** Characteristics of the respondents. **Appendix C.** Bivariate correlations between categories of risk factors. **Appendix D.** Perceived importance of risk factors by categories.

**Additional file 2.**

## Data Availability

All data are available on request to the corresponding author.

## Abbreviations

MAE: Medication administration error
ICU: Intensive Care Unit
HER: Electronic Health Record
BCMA: Barcode Medication Administration
DERS: Dose Error Reduction System
