# Supplementary material for: Medication administration errors in the domain of infusion therapy in intensive care units: a survey study among nurses
Source: Arch Public Health. 2023 Feb 15;81:23. doi: 10.1186/s13690-023-01041-2 (PMC9930049; doi:10.1186/s13690-023-01041-2)
Supplement: Supplementary file 1 — Additional file 1: Appendix A. Survey English version (translated). Appendix B. Characteristics of the respondents. Appendix C. Bivariate correlations between categories of risk factors. Appendix D. Perceived importance of risk factors by categories. [file 13690_2023_1041_MOESM1_ESM.docx]

Appendix

A) Survey English version (translated)

**A. Characteristics of the respondent and the hospital**

**What type of hospital do you work?**

1. General hospital
2. Academic hospital
3. Top-clinical hospital
4. Other:

**What is the size of the hospital you are working at in terms of ICU beds?**

1. 0-10
2. 11-20
3. 21-30
4. 31-40
5. 41-50

**My job function is:**

1. IC Nurse
2. Management position
3. Other:

**What is your gender?**

- 1. Female
  2. Male
  3. Other:

**What is your age?**

Open answer

**How many years of work experience do you have as nurse working on the ICU?**

Open answer

**B. Medication errors**

**What is the (estimated) number of medication errors that occur during drug administration of intravenous medication with infusion therapy on the ICU per week?**

Open answer

|  | **How often do you estimate that the listed errors occur in your ICU department during infusion therapy (regardless of whether they lead to consequences)** | | | | | **In your opinion, what is the estimated severity of consequences after the occurrence of the listed errors?** | | | | **To what extent do you think the listed errors are preventable?** | | | | | |
| --- | --- | --- | --- | --- | --- | --- | --- | --- | --- | --- | --- | --- | --- | --- | --- |
|  | Never | Annual | Monthly | Weekly | Daily | No consequences | Reversible consequences | Severe consequences | Very severe/deadly consequences | Very unpreventable | Unpreventable | Neutral | Preventable | Very preventable |  |
| Omission of medication |  |  |  |  |  |  |  |  |  |  |  |  |  |  |  |
| Administering the wrong medication |  |  |  |  |  |  |  |  |  |  |  |  |  |  |  |
| Wrong dose: under dose |  |  |  |  |  |  |  |  |  |  |  |  |  |  |  |
| Wrong dose: overdose |  |  |  |  |  |  |  |  |  |  |  |  |  |  |  |
| Wrong concentration |  |  |  |  |  |  |  |  |  |  |  |  |  |  |  |
| Wrong infusion rate |  |  |  |  |  |  |  |  |  |  |  |  |  |  |  |
| Incorrect duration of infusion administration |  |  |  |  |  |  |  |  |  |  |  |  |  |  |  |
| Wrong route |  |  |  |  |  |  |  |  |  |  |  |  |  |  |  |
| Administering medication to the wrong patient |  |  |  |  |  |  |  |  |  |  |  |  |  |  |  |
| Administration of medication at the wrong time (more than 1 hour different from time) |  |  |  |  |  |  |  |  |  |  |  |  |  |  |  |
| Incorrect setting of the infusion pump |  |  |  |  |  |  |  |  |  |  |  |  |  |  |  |
| Too late to intervene in the event of pump alarm |  |  |  |  |  |  |  |  |  |  |  |  |  |  |  |
| Not changing an infusion or syringe in time |  |  |  |  |  |  |  |  |  |  |  |  |  |  |  |
| Incorrect combination of medication |  |  |  |  |  |  |  |  |  |  |  |  |  |  |  |
| Medication administered in case of known allergy or intolerance of the patient |  |  |  |  |  |  |  |  |  |  |  |  |  |  |  |
| Administering a non-prescribed medication (without prescription) |  |  |  |  |  |  |  |  |  |  |  |  |  |  |  |
| Incorrect recording of administration in file |  |  |  |  |  |  |  |  |  |  |  |  |  |  |  |

**C. (Risk) factors**

**The next question is about (risk) factors. Here we are talking about factors that increase the occurrence of medication errors during the administration of medication via infusion therapy. To what extent do you think these factors are important for the occurrence of medication errors during infusion therapy in the ICU department?**

|  | **1 (very unimportant)** | **2** | **3** | **4 (neutral)** | **5** | **6** | **7 (very important)** |
| --- | --- | --- | --- | --- | --- | --- | --- |
| **Medication-related factors** | | | | | | | |
| Type of medication (effect) |  |  |  |  |  |  |  |
| Large amount of medication |  |  |  |  |  |  |  |
| No or incorrect dosage/concentration on labels on label |  |  |  |  |  |  |  |
| **Patient-related factors** | | | | | | | |
| Severity of the disease |  |  |  |  |  |  |  |
| Large number of treatments/interventions |  |  |  |  |  |  |  |
| Long-term hospitalizations |  |  |  |  |  |  |  |
| Sedation: patients unable to participate in care and defend themselves against mistakes |  |  |  |  |  |  |  |
| Lack of usual medication list (home medication) |  |  |  |  |  |  |  |
| Patient agitation |  |  |  |  |  |  |  |
| **Intensive care-related factors** | | | | | | | |
| Complex environment |  |  |  |  |  |  |  |
| Urgent admissions |  |  |  |  |  |  |  |
| Multiple different caregivers working |  |  |  |  |  |  |  |
| Initiation of temporary medication therapies |  |  |  |  |  |  |  |
| Use of new technologies and treatments |  |  |  |  |  |  |  |
| Deviation from protocols / hospital policy |  |  |  |  |  |  |  |
| Patient-nurse ratio on the ward |  |  |  |  |  |  |  |
| Frequent staff changes and frequent transfers of care |  |  |  |  |  |  |  |
| Insufficient supervision of the caregivers |  |  |  |  |  |  |  |
| Premature and nocturnal discharge |  |  |  |  |  |  |  |
| **Care professional-related factors** | | | | | | | |
| Shortage of work experience |  |  |  |  |  |  |  |
| Shortage of medication knowledge |  |  |  |  |  |  |  |
| Mental state/sleep deprivation/fatigue |  |  |  |  |  |  |  |
| Problems in communication between caregivers |  |  |  |  |  |  |  |
| Insufficient information and/or knowledge about using the pump |  |  |  |  |  |  |  |
| Wrong reading of the file |  |  |  |  |  |  |  |
| Notation errors in file |  |  |  |  |  |  |  |
| Shifts: early shift |  |  |  |  |  |  |  |
| Shifts: late shift |  |  |  |  |  |  |  |
| Shifts: night shift |  |  |  |  |  |  |  |
| **Infusion pump-related factors** | | | | | | | |
| Programming/choosing the right pump (software) |  |  |  |  |  |  |  |
| Problem in the pre-administration process |  |  |  |  |  |  |  |
| Hoses/connections |  |  |  |  |  |  |  |
| Infusion pump failure |  |  |  |  |  |  |  |
| Snapping the correct syringe into the pump |  |  |  |  |  |  |  |
| Device maintenance |  |  |  |  |  |  |  |
| Insufficient presence of required characteristics of the pump |  |  |  |  |  |  |  |
| Low user-friendliness |  |  |  |  |  |  |  |

**Which risk factors are also important for the occurrence of medication errors, and would you add to the list?**

Open answer

**D. Infusion pump**

|  | **Do the pumps in your ICU department have the following features?** | | | **In your opinion, what is the importance of the following features in reducing the risk of medication errors?** | | | | | | |
| --- | --- | --- | --- | --- | --- | --- | --- | --- | --- | --- |
|  | Yes | No | I don’t know | 1 (very unimportant) | 2 | 3 | 4 (neutral) | 5 | 6 | 7 (very important) |
| Pre-programmed protocols for minimum and maximum dose |  |  |  |  |  |  |  |  |  |  |
| Pre-programmed protocols for the concentration/dilution ratios |  |  |  |  |  |  |  |  |  |  |
| Pre-set start speed and minimum / maximum speed that considers the weight of the patient |  |  |  |  |  |  |  |  |  |  |
| Locking systems and alarm systems in case of wrong dose setting |  |  |  |  |  |  |  |  |  |  |
| Ability to remotely monitor and modify the infusion |  |  |  |  |  |  |  |  |  |  |
| Ability to set highly accurate occlusion pressure parameters |  |  |  |  |  |  |  |  |  |  |
| Ability to load the syringe (or bag) and set the infusion parameters quickly in case of emergencies |  |  |  |  |  |  |  |  |  |  |
| Infusion rate control systems |  |  |  |  |  |  |  |  |  |  |
| In the event of an emergency start, the possibility to enter all safety procedures later |  |  |  |  |  |  |  |  |  |  |
| Smart pumps with Dose Error Reduction Software (DERS) |  |  |  |  |  |  |  |  |  |  |
| Pre-programmed protocols for minimum and maximum dose |  |  |  |  |  |  |  |  |  |  |

**E. Information systems**

**Is administration with the infusion pump electronically recorded in your ICU department?**

1. Yes
2. No
3. I don’t know

**To what extent do you think that electronic recording of administration is important for reducing medication errors?**

1. 1 (very unimportant)
2. 2
3. 3
4. 4 (neutral)
5. 5
6. 6
7. 7 (very important)

**Optional: what is the current practice if you do not use an electronic control system for administration?**

Open answer

**Does the electronic system check the right patient and the right medication before administration by means of barcode readers and/or optical medication scanners?**

1. Yes
2. No
3. I don’t know

**To what extent do you think the use of barcode readers and/or optical medication scanners is important for reducing medication errors?**

1. 1 (very unimportant)
2. 2
3. 3
4. 4 (neutral)
5. 5
6. 6
7. 7 (very important)

**Are the infusion pumps in your department equipped with software that allows you to create anonymous statistical reports that identify all the setting errors that are committed?**

1. Yes
2. No
3. I don’t know

**Optional: How often are reports – that mention setting errors – analyzed?**

1. Never
2. Annual
3. Monthly
4. Weekly
5. Daily

**In your opinion, what is the importance of anonymous reports to analyze any errors in the use and setting of the infusion pump in order to reduce the number of medication errors?**

1. 1 (very unimportant)
2. 2
3. 3
4. 4 (neutral)
5. 5
6. 6
7. 7 (very important)

**Are any medical devices (vital signs monitors, smart pumps etc.) currently connected to your hospital’s EHR?**

1. Yes
2. No
3. I don’t know

**To what extent do you think that this connectivity of medical devices (via the EHR) is important for reducing medication errors?**

1. 1 (very unimportant)
2. 2
3. 3
4. 4 (neutral)
5. 5
6. 6
7. 7 (very important)

**F. Feedback culture and reporting**

**Are error reports accessible to you?**

1. Yes
2. No
3. I don’t know

**How often do you consult these error reports?**

1. Never
2. Annual
3. Monthly
4. Weekly
5. Daily

**Do you think error reports are important for reducing medication errors in your ICU department?**

1. 1 (very unimportant)
2. 2
3. 3
4. 4 (neutral)
5. 5
6. 6
7. 7 (very important)

B) Characteristics of the respondents

|  | Frequencies  N, % | Mean ± SD | Median (IQR) |
| --- | --- | --- | --- |
| Sex  Women  Men | 53 (58.2)  38 (41.8) |  |  |
| Age (years) |  | 43.8 ± 11.7 | 44 (32-54) |
| Years of experience |  | 16.7 ± 11.2 | 15 (5.5-25.5) |
| Type of hospitals  General hospital  Academic hospital  Top-clinic hospital* | 46 (50.4)  22 (24.2)  22 (24.2) |  |  |
| Size of ICUs  ≤10 beds  11-20 beds  21-30 beds  31-40 beds  40-50 beds | 10 (11)  32 (35.2)  24 (26.4)  19 (20.9)  6 (6.6) |  |  |

SD: standard deviation ; IQR: interquartile range

*Top-clinic hospitals in the Netherlands are a type of non-university hospital that offer a level and type of care similar to that of university hospitals. Because they are not directly affiliated with a university, these hospitals tend to be somewhat smaller.

C) Bivariate correlations between categories of risk factors

|  | **Medication-related factors** | **Patient-related factors** | **Intensive care-related factors** | **Care professional-related factors** | **Infusion pump-related factors** |
| --- | --- | --- | --- | --- | --- |
| **Medication-related factors** | . | r=0.44, p<0.001 | r=0.32, p=0.002 | r=0.39, p<0.001 | r=0.35, p<0.001 |
| **Patient-related factors** |  | . | r=0.49, p<0.001 | r=0.30, p=0.004 | r=0.55, p<0.001 |
| **Intensive care-related factors** |  |  | . | r=0.48, p<0.001 | r=0.61, p<0.001 |
| **Care professional-related factors** |  |  |  | . | r=0.63, p<0.001 |
| **Infusion pump-related factors** |  |  |  |  | . |

D) Perceived importance of risk factors by categories

|  | **n** | **Medication-related factors** | **Patient-related factors** | **Intensive care-related factors** | **Professional-related factors** | **Infusion pump-related factors** |
| --- | --- | --- | --- | --- | --- | --- |
| **Age**  < 45 years  ≥ 45 years  p-value | 46  45 | 5.2±1.19  5.1±1.15  0.78 | 4.42±1.28  4.63±1.18  0.43 | 4.88±0.87  5.13±0.75  0.15 | 5.35±0.96  5.16±1.03  0.40 | 4.15±1.28  4.27±1.37  0.68 |
| **Sex**  Women  Men  p-value | 53  38 | 5.06±1.09  5.31±1.26  0.31 | 4.51±1.21  4.55±1.27  0.86 | 4.98±0.82  5.04±0.83  0.75 | 5.27±0.94  5.24±1.09  0.91 | 4.33±1.21  4.04±1.46  0.32 |
| **Experience**  < 15 years  ≥ 15 years  p-value | 43  48 | 5.21±1.18  5.13±1.16  0.75 | 4.39±1.30  4.64±1.16  0.34 | 4.91±0.90  5.09±0.73  0.31 | 5.44±0.96  5.08±1.00  0.09 | 4.28±1.29  4.14±1.36  0.62 |
| **Number of ICU beds**  0-20 beds  ≥20 beds  p-value | 42  49 | 5.07±1.08  5.25±1.23  0.45 | 4.49±1.08  4.55±1.35  0.81 | 5.05±0.67  4.96±0.93  0.64 | 5.18±0.92  5.36±1.06  0.48 | 4.36±1.14  4.08±1.46  0.29 |
| **Type of hospital**  General  Academic  Top  p-value | 46  22  22 | 5.35±1.05  4.89±1.39  5.03±1.15  0.26 | 4.75±1.22  4.19±1.39  4.34±0.98  0.16 | 5.05±0.85  4.80±0.74  5.08±0.84  0.43 | 5.41±0.96  4.98±1.19  5.17±0.83  0.24 | 4.52±1.33  3.52±1.26  4.21±1.14  0.01 |

Level of significance was calculated using Student T test for Age, Sex, Experience and number of ICU beds and using ANOVA test for Type of hospital
